# Effectiveness of prophylaxis treatment in the acute febrile stage of febrile seizure in children under five years old 

**DOI:** 10.22037/ijcn.v15i4.18740

**Published:** 2022-01-01

**Authors:** Afshin FAYYAZI, Nasrollah PEZESHKI, Firoozeh HOSSEINI, Reyhane ESLAMIAN, Farzaneh ESNAASHARI

**Affiliations:** 1 Department of Pediatrics, School of Medicine, Hamadan University of Medical Sciences, Hamadan, Iran; 2 Department of Community Medicine, School of Medicine, Hamadan University of Medical Sciences, Hamadam, Iran.

**Keywords:** Children, Febrile seizure, Prophylaxis, Diazepam, Phenobarbital

## Abstract

**Objectives:**

In children suffering from febrile seizure, the likelihood of recurrence seems to be high in the early hours following the first episodes in the absence of proper interventions. The present study was aimed at assessing and comparing the outcomes of different preventive interventions in the acute stage after febrile seizure in children.

**Materials & Methods:**

This randomized clinical trial study was performed between September 2015 and September 2016. We enrolled patients aged between 6 and 60 months suffered from febrile seizure and referred to the Pediatric Emergency Department at Besat Hospital in Hamadan. The eligible patients were randomly assigned to the following four receive one of the following groups: group 1 (not receiving any anti-seizure drugs), group 2 (receiving a single dose of phenobarbital) on admission, group 3 (receiving a single dose of phenobarbital on admission continued until the fever is resolved), and group 4 (receiving diazepam until the disappearance of fever).

**Results:**

The study population consisted of 248 children. The recurrence rate of seizure in the acute stage was 4.84%. Also, the impact of diazepam and phenobarbital (either as a single dose or as continuous) on the prevention of febrile seizure recurrence in the acute stage has been established. None of the patients had febrile status epilepticus.

**Conclusion:**

Controlling seizures without prescribing anti-seizure drugs increases the risk for the recurrence of febrile seizure in the acute stage. Different drug regimens for controlling seizure, including diazepam and phenobarbital (as stat or maintenance), may play a similar role in preventing the occurrence of febrile seizure.

## Introduction

Febrile convulsions (FC) denote seizures occurring in febrile children aged between the ages of 6 and 60 months who do not have an intracranial infection, metabolic disturbance, or history of afebrile seizures. Febrile seizures are subdivided into the two categories of simple and complex. Simple febrile seizures last for less than 15 minutes, are generalized (without a focal component), and occur once in a 24-hour period, whereas complex febrile seizures are prolonged (15 minutes) and focal or occur more than once in 24 hours (1). 

Despite the frequency of febrile seizures (2%–5%), there is no unanimity of opinion regarding management options (1,2, 3,4). When a single FC or repetitive FC last more than 30 min without the recovery of the normal consciousness, a febrile status epilepticus occurs (5). Experimental studies performed in the 80s showed that the central nervous system (CNS) homeostasis was constitutively altered in the case of seizures lasting more than 30 min (6). In this regard, the literature suggests that short seizure duration may reduce CNS risk factors. On the other hand, further studies showed that a single epileptic event usually resolves within 2–5 min, while those critical events lasting more than 10–15 min show a higher risk and are not spontaneously resolved (7). Although this phenomenon is naturally benign, it may lead to severe anxiety among parents (8). Besides its benign nature, the high likelihood of some serious complications, such as status epilepticus and mesial temporal sclerosis, highlight the importance of the early management of febrile seizure (9). 

In this regard, recurrent febrile seizure is primarily critical. Several factors have been identified to increase the risk for recurrent febrile seizure, including age less than one year at the time of the first seizure, family history of febrile seizure in relatives, low fever during seizure, and the occurrence of complex seizures (10,11). The optimal management of febrile seizure is mainly based on three steps, including 1) management of the patient at the time of seizure and status epilepticus, 2) management of the recurrence of febrile seizure in the early hours after seizure interruption, and 3) management of the recurrence of febrile seizure in the next febrile courses (12,13). In the first step, the treatment protocol is similar to the seizure conditions without fever. In subsequent steps, the early prevention of seizure can result in favorable outcomes. Due to the benign nature of this event, it is not necessary to follow an especial preventive or therapeutic protocol, except for those conditions with a high risk for recurrent episodes or stress in parents (14). Moreover, in the third step, two preventive approaches are now accepted, including intermittent treatment with oral diazepam only during the onset of fever and long-term treatment with phenobarbital or sodium valproate daily (15). In total, and according to some evidence, the likelihood of recurrence is high in the early hours in the absence of proper interventions (16). Furthermore, using various preventive interventions may lead to different outcomes in affected children. The present study was aimed at assessing and comparing the outcome of different preventive interventions in the early hours after febrile seizure. The aim of this study was to evaluate the effect of prophylaxis treatment in acute FC stage until fever subsides.

## Materials & Methods

This randomized clinical trial was conducted among children aged between 6 and 60 months and suffered from febrile seizure who referred to the Pediatric Emergency Department at Besat Hospital in Hamadan between September 2014 and September 2015. The inclusion criteria were the occurrence of simple febrile seizure for the first time, complex febrile seizure (up to two times) defined as seizures lasting >15 min and/or focal and/or repetitive within 24 h and/or with postictal neurological abnormalities, recurrent febrile seizure up to two times, the presence of fever (higher than 37.5ºC axillary at the time of seizure), no history of previous seizures without fever, and absence of a neurological development disorder or a previous lesion of the brain. The exclusion criteria comprised of status epilepticus, change in the diagnosis, including meningitis or encephalitis, the presence of electrolyte disturbances in seizure, including hypoglycemia and hypocalcemia, lack of parental cooperation, serial seizures before admission to the hospital (more than twice), any abnormalities in brain imaging, allergy to phenobarbital manifested by rash, or Stevens-Johnson syndrome or TEN. The eligible patients were randomly (using the block randomization method) assigned to receive one of the following protocols: group 1 (not receiving any anti-seizure drugs), group 2 (receiving a single dose of phenobarbital (10 mg/kg, IM) on admission), group 3 (receiving a single dose of phenobarbital (10 mg/kg, IM) on admission followed by 2.5 mg/kg/12 hour, IM and then beginning of oral regimen as phenobarbital (2.5 mg/kg, OR) until fever subsided, and group 4 (receiving Tab diazepam (0.33 mg/kg/q8h) until fever was gone).

In cases with the recurrence of seizure (regardless of the study group), phenobarbital as 10 mg/kg was administered IM followed by 2.5 mg/kg/12h intramuscularly. In cases with status epilepticus (more than 30 minutes or two consecutive seizures with lack of consciousness among seizures lasting more than 30 minutes), the routine protocol for treating status epilepticus was followed. All the patients were evaluated with regard to the cause of fever. In cases with uncontrolled fever, acetaminophen (10 to 15 mg/kg) in the form of syrup was administered. Supplementary studies, including laboratory analyses and CSF analysis or imaging, were planned if required.

Results are presented as mean ± standard deviation (SD) for quantitative variables and as frequency (percentage) for categorical variables. Continuous variables were compared using ANOVA or Kruskal-Wallis test whenever the data did not appear to have a normal distribution or when the assumption of equal variances was violated across the study groups. Categorical variables, on the other hand, were compared using the Chi-square test. A *P*-value of less than 0.05 was considered statistically significant, SPSS version 23.0 for windows (IBM, Armonk, New York) was used for data analysis.

## Results

In total, 248 patients (129 male and 119 female, mean age: 22.00 ± 10.98 months, age range: 6 to 60 months) were included. Regarding drug-related regimens for controlling seizure, 68 patients were considered as the control without planning for anti-seizure medication, 63 patients received diazepam, 59 patients received a single dose of phenobarbital, and 58 patients were planned for treatment with continuous phenobarbital administration. 

Overall, 95.6% of the patients had no recurrent seizure, and 4.84% the was recurrence rate of FC (4.4% experienced seizure once and only 0.4% experienced it twice). Recurrence rate was 13.2% (11.8% had one episode and 1.5% had two episodes) in the non-drug group, 3.2% in the diazepam group, and 1.7% in the single dose of phenobarbital group (P=0.000; Table 1). None of the patients had febrile status epilepticus.

Regarding the type of seizure, simple seizure was found in 79.4% of the patients, complex in 5.2 %, and recurrence in 15.3%. The mean of body temperature was measured to be 38.64 degrees (range: 37 to 41); specifically, 107 patients had fever below 38.5 degrees and 141 patients had fever above 38.5 degrees. The main causes of fever were respiratory infectious disorders (86.7%), gastrointestinal infections (9.7%), urinary tract infections (0.8%), and other infectious conditions (2.8%). Family history of seizure and febrile seizure was found in 14.1% and 26.6% of the cases, respectively. The mean time between the appearance of fever and seizure was 35.19 hours; in detail, in 203 patients it was less than 24 hours, and in 45 cases it was more than 24 hours. None of the patients had hyponatremia, and we found no relationship between the frequency of seizure (p=0.36) and the patients' gender (p = 0.874), age (p=0.814), type of seizure (p = 0.396), body temperature (p = 0.643), cause of febrile seizure (p = 0.051), family history of febrile seizure (p = 0.729), family history of seizure (p = 0.355), and time between fever and seizure (p = 0.211). 

## Discussion

This study was conducted at the Department of Pediatrics during September 2015 and September 2016. The purpose of this study was to evaluated the effect of prophylaxis treatment in the acute febrile stage after the FC in children. The use of diazepam and phenobarbital (either as a single dose or as continuous ) were effective in preventing the recurrence of FC in the acute stage. Diazepam and a single dose of phenobarbital were 4.5 and 8.7 times more effective than non-drug use in preventing the recurrence of FC in the acute stage.

It was also found that FC recurrence was not significantly associated with age, sex, recurrence, rate, duration of fever to seizure, type of FC, cause of fever, family history of FC, family history of epilepsy, hyponatremia, and preservation in kindergarten. 

In the present, the recurrence rate of FC in the acute stage was 4.84%. Barzegar et al. (16) in 2009 reported that recurrence rate in the acute stage was 18.6% in a no drug group and 2.8% in oral and intravenous phenobarbital groups. In 2006, Barzegar et al. (17 ) concluded that recurrence rate in the acute phase was 15% in a no drug group and 1% in a phenobarbital group. In the study of Hirabayashi et al. (18), 203 children with FC were included. They were given diazepam suppository in the acute phase of illness, while the control group did not receive any drugs. It was reported that the recurrence rate was 2.1% in the diazepam group, and 14.8% in the no-drug group. It can be stated that the only predictor of the recurrence of FC in the acute stage in our study was the type of treatment protocol. Although FC is a benign phenomenon, since most studies report the highest rate of recurrence in the first year and in the first 24 hours, administering drugs to the child in the acute stage of FC has more advantages than disadvantages.

In the present study, the mean age of the patients was 22 months, which was comparable to participants enrolled by Nelson et al. study (1), where the mean age was 23.3 months, and Manish et al. (19 ) and Fallah et al. studies (20), where the mean age was 26.72 and 24.3 months, respectively. All of these studies showed a significant relationship between age at FC and recurrence. Similar to our study, Ojha et al. (21) did not prove such a relationship.

In the present study, the mean interval between fever onset and seizure occurrence was 19.35 hours. Hence, there was no significant association between the recurrence of FC and duration of fever. Similarly, the studies conducted by Chan et al. (22) and Fallah et al. showed a significant link between recurrent FC and shorter duration of fever before onset of seizure.

In our study, the prevalence of FC was higher in boys, but there was no significant relationship between gender and recurrence. This result was consistent with the results of Fallah et al. and Chan et al., but it was not compatible with the findings of Bessisso et al. (23) and Agrawal et al. (24).

In the present study, the family history of FC and epilepsy was present in one-third and one-seventh of the subjects. The association between the family history of FC or epilepsy and the recurrence was not found to be statistically significant, which is similar to the results of Talebian et al., Manish et al., and Ojha et al. However, Nelson et al. and Berg et al. (25 ) showed a strong association between family history and recurrence. The genetic contribution to the incidence of FC was manifested by a positive family history of FC in several patients. In some families, the disorder was inherited as an autosomal dominant trait, and the multiple and single genes causing the disorder appear to be polygenic, but the predisposing genes remain to be identified.

The type of seizure of the patients was simple, which is consistent the studies of Imani et al. (26) and Salehi (27), but in the study by Agrawal (24) most patients had complex FC; this discrepancy could be due to geographical and race differences.

In the present study, the highest rate of recurrence was in recurrent FC, followed by simple and complex FC. Therefore, there was no significant association between recurrence and type of seizure, which is consistent with the findings of Manish et al., Chan et al., Fallah et al., and Berg et al. However, Ojha et al. and Leung et al. (28) found a significant association between the complex type of seizure and FC recurrence. Considering that complex FC is a minor risk factor for the recurrence of seizure, it seems that the reason for the low recurrence rate in our study is a short follow-up period (in the acute stage). Race and geographical differences can also account for the low prevalence of complex FC in our study. A similar study with a larger sample size is suggested to corroborate our findings. In the present study, the most important causes of fever included upper respiratory infection, gastroenteritis, UTI, and other infectious causes, respectively.

**Table 1:** Recurrence frequency of febrile seizure in four groups

		Recurrence frequency			total
		No		Yes	
Type of drug	No drug	59	9		68
		86/8%	13/2%		100%
	Tab diazepam	61	2		63
		83/96%	17/3%		100%
	Phenobarbital (single dose)	57	1		58
		98/3%	1/7%		100%
	Phenobarbital(continuous)	59	0		59
		100%	0%		100%
Total		236	12		248
		95/16%	4/84%		100%

P =000.0

## Conclusion

 Controlling seizures without prescribing anti-seizure drugs increases the risk for the recurrence of febrile seizure in the acute stage. Different drug regimens for controlling seizure, including diazepam and phenobarbital (as stat or maintenance), may play a similar role in preventing the occurrence of febrile seizure. 

## Author Contribution

Afshin Fayyazi: literature search; study concepts and design ,manuscript preparation, Ali Khajeh:, manuscript editing and review.

Reihane Eslamian : clinical studies, data acquisition ,analysis, statistical analysis;

## Conflict of interest

None
